# PAX8 Distinguishes Diffuse Large B-Cell Lymphoma Mimicking Sarcoma

**DOI:** 10.1155/2017/6714549

**Published:** 2017-05-28

**Authors:** Michelle S. Hirsch, Alessandra F. Nascimento

**Affiliations:** ^1^Department of Pathology, Brigham and Women's Hospital and Harvard Medical School, Boston, MA, USA; ^2^Diagnose Anatomia Patologica e Citopatologia, Rio de Janeiro, RJ, Brazil

## Abstract

*PAX8* is important for embryogenesis of the thyroid, Müllerian system, and upper urinary/renal tract, and expression of PAX8 has been described in carcinomas from each of these sites. The sensitivity and specificity of the polyclonal PAX8 antibody in a large cohort of epithelial tumors as well as lymphomas have been previously determined, the latter because polyclonal PAX8 is known to be immunoreactive in nonneoplastic B-cell lymphocytes which are often used as the positive internal control for immunohistochemistry. In this case report, PAX8 was a diagnostic clue for revising a previous diagnosis of unclassified high grade sarcoma to diffuse large B-cell lymphoma. This case report demonstrates a pitfall for PAX8 immunoreactivity and acts as a reminder that lymphoma should be included in the differential diagnosis of a PAX8 positive, epithelial cell marker negative tumor of unknown primary origin.

## 1. Introduction

A* Pax* gene contains a paired-box, DNA binding domain of 128 amino acids at the amino terminus. It is conserved among many species, and to date nine members of the* Pax *gene family have been identified. Distinction among the different* Pax* genes is dependent on the presence or absence of octapeptide coding regions and a paired type homeobox [1]. Individual members of the* Pax* gene family have been shown to be crucial for morphogenesis, organogenesis, cell differentiation, and/or tumorigenesis via their corresponding protein products, which act to regulate transcription [2].

PAX8 is a member of this paired-box family of genes and is expressed during organogenesis of the thyroid gland, Müllerian tract, and kidney [3, 4]. It is a 48 kD protein, encoded at locus 2q12-14, which was first described in the developing mouse thyroid gland [5]. It was later implicated in thyroid carcinogenesis, as a* PAX8-PPARgamma* rearrangement is found in ~30% of follicular carcinomas; however, this gene rearrangement is not observed in other subtypes of thyroid carcinoma [4, 6]. Nevertheless, clinical studies have shown that PAX8 protein expression is present in nearly all papillary and follicular carcinomas, whereas only a subset of anaplastic and medullary carcinomas is positive [7].

PAX8 has also been shown to be a lineage specific protein in both the kidney and the Müllerian tract [5]. PAX8, together with PAX2, regulates branching morphogenesis and nephron differentiation, whereas female mice lacking PAX8 demonstrate defects in genital tract formation and are unable to produce offspring [8]. Consistent with the role of PAX8 as a lineage specific marker, it has been shown to be expressed in benign epithelial structures and corresponding malignancies from the human kidney and Müllerian tract [9]. Clinically, PAX8 has been used to diagnose carcinomas of renal and Müllerian origin, especially when presenting as a metastasis, and to distinguish serous ovarian carcinoma from breast carcinoma, malignant mesothelioma, and primary adnexal tumors [9]. In addition to the aforementioned sites, PAX8 expression has also been reported in pancreatic endocrine tumors, a subset of renal pelvis urothelial carcinomas, clear cell adenocarcinomas of the bladder, and thymic neoplasms (see Laury et al., 2011, for additional references [9]).

Two series have addressed the expression of PAX8 in specific subtypes of lymphoma [2, 10]. PAX8 is known to be expressed in nonneoplastic B-lymphocytes, and these cells are frequently used as positive internal controls for immunohistochemistry [9]. Accordingly, polyclonal PAX8 (but not monoclonal PAX8) has been shown to be a sensitive and relatively specific marker of B-cell lymphomas (versus non-B-cell lymphomas) due to cross reaction with PAX5/B-cell specific activation protein (BSAP) [2, 10, 11]. Lymphoma and sarcoma are not frequently confused, but herein we report a case where PAX8 was able to diagnose a spindle cell variant of diffuse large B-cell lymphoma that had been previously diagnosed as an unclassified sarcoma.

## 2. Case Report/Pathologic Findings

**Figure 1:**
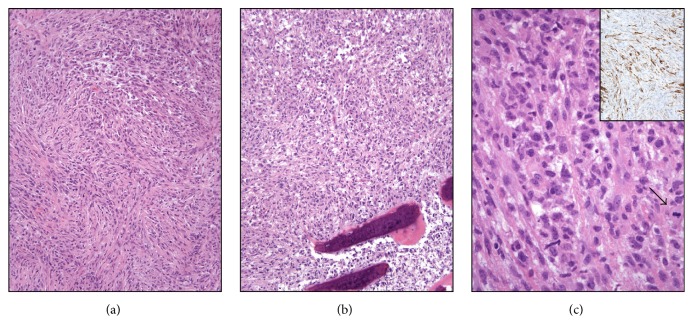
Morphologic features of the original dura/calvarium lesion. (a–c) H&E staining demonstrates a malignant spindle and epithelioid cell neoplasm with increased mitotic activity ((c), arrow) and necrosis (not shown). A large panel of immunostains, including markers of both epithelial and mesenchymal differentiation, was negative, except for focal expression of SMA (inset).

**Figure 2:**
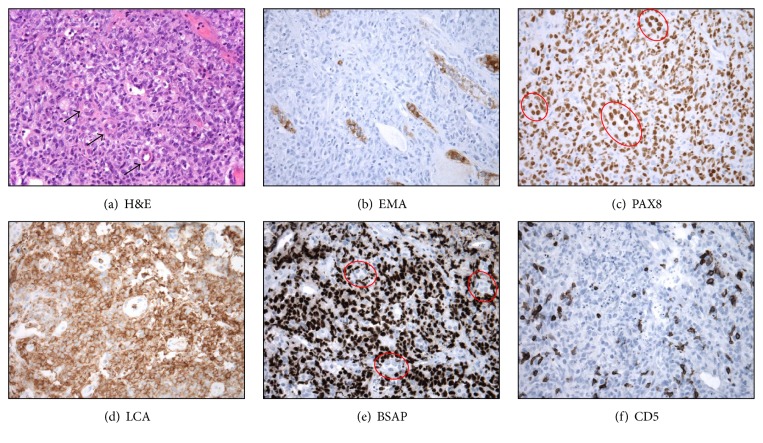
Morphologic and immunohistochemical features of the renal neoplasm support a diagnosis of diffuse large B-cell lymphoma. (a) H&E staining demonstrates an epithelioid neoplasm with a distinctive infiltrative pattern between renal tubules (nonneoplastic renal tubules highlighted by arrows). (b) An EMA immunostain is immunoreactive in the nonneoplastic renal tubules, but negative in the neoplasm. (c) A PAX8 immunostain is diffusely and strongly positive in both nonneoplastic renal tubules (red ovals) and the infiltrative neoplasm. (d–f) Subsequent LCA, PAX5/BSAP, and CD5 immunostains support the diagnosis of diffuse large B-cell lymphoma. Note the absence of PAX5 in nonneoplastic renal tubules (red ovals) and the presence of CD5 in nonneoplastic T-cell lymphocytes.

**Figure 3:**
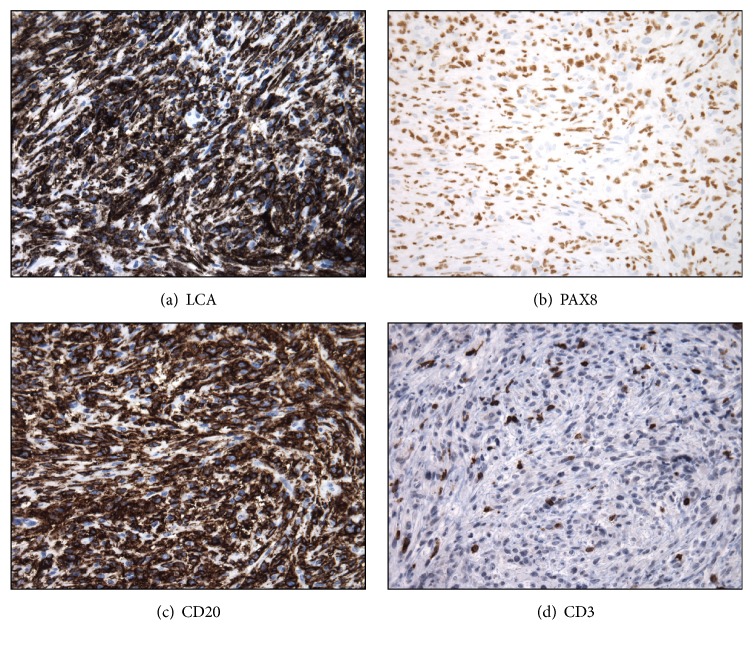
Reevaluation of the original dura/calvarium lesion is consistent with diffuse large B-cell lymphoma. (a-b) Immunostains for LCA and PAX8 were diffusely and strongly immunoreactive in the tumor, supporting the diagnosis of lymphoma. The presence of CD20 and the absence of CD3 in neoplastic cells were consistent with a lymphoma of B-cell lineage (CD3 is present in nonneoplastic T-cell lymphocytes).

A 71-year-old female patient first presented with painful nodules on her scalp in the frontal-parietal region in the spring of 2009. MRI and subsequent CT scans demonstrated frontal bone and adjacent midline subgaleal soft tissue abnormalities with no abnormality in the brain parenchyma. In January 2010, a frontal bone craniotomy and dural biopsy were performed. Pathology revealed a malignant spindle and epithelioid cell neoplasm (Figure 1) that was focally positive for smooth muscle actin (SMA) and negative for multiple keratins, EMA, CD34, Desmin, GFAP, and S100 (see Table 1 for antibody information). Focal necrosis was present and mitoses numbered up to 5 per 10 HPFs. The overall findings were felt to be most consistent with an unclassified high grade sarcoma. A PET scan performed in mid-February at a referring institution reported FDG uptake only in the scalp. Multiple CBCs over the course of the patient's workup were within normal limits. The following month, the patient began treatment with temozolomide and radiation therapy; the former was discontinued within one month secondary to profound thrombocytopenia, and the latter was continued for 3 months. A follow-up PET scan 5 months later was significant for FDG uptake in foci of both kidneys; the calvarium and brain parenchyma were unremarkable. A diagnostic biopsy of one kidney was performed which demonstrated an epithelioid neoplasm with a prominent infiltrative pattern between tubules (Figure 2(a)). The tumor was immunoreactive (Table 1) with PAX8 (Figure 2(c)), but negative for multiple keratins, EMA (Figure 2(b)), CD34, RCC antigen, SMA, and Desmin. Despite the presence of PAX8, a marker frequently used to confirm renal primary epithelial neoplasm, the infiltrative pattern as well as the cytology and lack of other markers typically seen in renal primaries raised the possibility of a B-cell lymphoma (PAX8 has been documented to stain nonneoplastic B-cells [9]). A subsequent round of immunohistochemistry (see Table 1 for antibody information) demonstrated that the neoplastic cells were positive for LCA (Figure 2(d)), CD20, PAX5/BSAP (Figure 2(e)), BCL6, and BCL2 (focal) and negative for CD3 and CD5 (Figure 2(f)). These findings confirmed the diagnosis of diffuse large B-cell lymphoma of follicular center cell derivation. In light of the kidney biopsy findings, the slides from the cranial/dural lesion were rereviewed, and a mixed epithelioid and spindle cell population was confirmed (Figure 1). Although spindle cell morphology is an uncommon finding in diffuse large B-cell lymphoma, the presence of morphologic overlap between neoplastic epithelioid cells in the two specimens warranted further work. Subsequent immunostains on the dura specimen demonstrated the presence of PAX8, LCA, and CD20 and an absence of CD3 in both the spindle and the epithelioid cell components (Figure 3), confirming the diagnosis of diffuse large B-cell lymphoma, spindle cell variant. The patient was transferred to hematology-oncology physicians for further workup and treatment.

## 3. Discussion

Non-Hodgkin's lymphomas (NHL) are a heterogeneous group of neoplasms arising from B-, T-, or natural killer lymphocytes, accounting for approximately 3% of all adult neoplasms. Mature B-cell neoplasms compose the vast majority of NHL and account for more than 80% of the neoplasms in this group. Subsequent classification of B-cell lymphomas is based on the cytologic, architectural, and immunophenotypic characteristics of the neoplastic cells.

Diffuse large B-cell lymphoma (DLBCL) can be further subclassified into several entities with distinct clinicopathologic characteristics. The most common variant is that of DLBCL not otherwise specified (NOS). It more commonly affects middle-aged and older adults with a slight male predominance but may also be seen in children and young adults. It is ubiquitous in its site of involvement, occurring in virtually any nodal or extranodal location. Patients often present with painless, rapidly growing, large tumor masses.

Morphologically, DLBLC is characterized by the proliferation of large polygonal cells with irregular and convoluted nuclear contours, vesicular chromatin, variably prominent nucleoli, and scant amount of pale to eosinophilic cytoplasm. Occasionally, tumors may show myxoid change or anaplastic features. Rarely, neoplastic cells may show elongated cytomorphology and be arranged in fascicles or a storiform manner, mimicking a spindle cell neoplasm, such as a spindle cell sarcoma or sarcomatoid carcinoma. Females and males appear equally affected, with a predominance in middle-aged and older patients [12–14]. Similar to DLBCL-NOS, spindle cell variants of DLBCL appear to be widely distributed, including skin, liver, soft tissues, salivary glands, mediastinum, and female genital tract [12–14]. Concurrent lymph node involvement is seen only in a subset of cases. In most reported cases, the spindle cell variant of diffuse large B-cell lymphoma is positive for the B-cell marker CD20 with coexpression of bcl-6 in a subset of reported cases, consistent with follicular center cell derivation [12–14]. Up to 56% of cases with spindle cell morphology have been reported to express at least focal smooth muscle actin (SMA) [12, 13, 15].

Given the rarity of spindle cell variant of DLBCL, the diagnosis may be difficult for the general surgical pathologist. Therefore, the application of immunohistochemical stains is of pivotal importance for the correct diagnosis. Interestingly, in the case herein described, the diagnosis of lymphoma was only first entertained after neoplastic cells in the kidney demonstrated nuclear expression of PAX8, a marker known to immunoreact with renal epithelial neoplasms.

PAX8 is encoded by a gene in the* Pax* family. The genes in this family play important roles in morphogenesis, organogenesis, cell differentiation, and tumorigenesis [5]. Expression of PAX8 has been shown to be relatively limited to specific tissue types, including thyroid, kidney, and Müllerian epithelium, and it is a helpful immunostain for neoplasms arising in these sites [9]. Furthermore, PAX8 has been observed to be expressed in small, nonneoplastic B-cell lymphocytes as well as lymphomas of B- but not T-cell lineage [2, 9, 10].

PAX5 protein, another transcription factor encoded by a gene in the* Pax* family, also known as B-cell specific activation protein (BSAP), has been demonstrated to be crucial for B-cell commitment and function [16]. This marker is now routinely used in hematolymphoid cases and has been shown to be positive in cases of B-lymphoblastic leukemia, NHL of B-cell origin lacking plasmacytic differentiation, and cases of classical Hodgkin's lymphoma [11, 17, 18]. Importantly, this marker remains positive in cases of lymphoma in which patients have been treated with rituximab, a drug which targets the CD20 molecule. Additionally, this marker can also be useful in the diagnosis of rare cases of poorly differentiated B-cell neoplasms that may show loss of expression of other markers such as CD20 and leukocyte common antigen (LCA).

In association with the findings described in the literature as well as the observations herein described, we reinforce that PAX8 can, by cross reacting with PAX5, be a useful marker for leukemias and lymphomas of B-cell derivation. Additionally, the findings in this report demonstrate a potential pitfall for PAX8 immunoreactivity and suggest that B-cell lymphoma should be considered in the differential diagnosis when a tumor of unknown origin is immunoreactive for PAX8, but negative for epithelial or other specific sarcoma markers.

## Figures and Tables

**Table 1:** Immunohistochemistry: sources, clones, and dilutions.

Antibody	Source	Clone or catalogue #	Dilution
BCL2	Abcam	Clone E17	1 : 200
BCL6	Dako	Clone PG-B6p	1 : 25
CD3	LabVision	Clone SP7	1 : 300
CD5	Leica	Clone 4C7	1 : 400
CD20	Dako	Clone L26	1 : 500
CD34	BD Biosciences	Clone my10	1 : 40
CD45 (LCA)	Dako	Clone 2B11 + PD7/26	1 : 500
CK (pankeratin)	Dako	Clone MNF-116	1 : 700
Desmin	Sigma	Clone D33	1 : 500
EMA	Dako	Clone E29	1 : 200
GFAP	Dako	Polyclonal (catalogue #Z033429-2)	1 : 15,000
PAX5/BSAP	BD Biosciences	C = clone 24/PAX-5	1 : 200
PAX8	Proteintech Group	Polyclonal (catalogue # 10336-1-AP)	1 : 1200
RCC	Dako	Clone SPM314	1 : 400
SMA	Sigma	Clone 1A4	1 : 20,000
